# Patients and physiotherapy professionals’ perspectives on group-based treatments: a systematic review protocol

**DOI:** 10.1136/bmjopen-2024-095201

**Published:** 2025-06-03

**Authors:** Mélanie Le Berre, Maude Laberge, Farzaneh Yousefi

**Affiliations:** 1 Faculté de médecine, Université Laval, Quebec City, Quebec, Canada; 2 VITAM Centre de Recherche en Santé Durable, Quebec, Quebec, Canada; 3 Centre de recherche du CHU de Québec-Université Laval Site CHUL, Quebec, Quebec, Canada; 4 Centre interuniversitaire de recherche en analyse des organisations, Montreal, Quebec, Canada; 5 Department of Health Management, Policy, and Economics, Faculty of Management and Medical Information Sciences, Kerman University of Medical Sciences, Kerman, The Islamic Republic of Iran

**Keywords:** Physical Therapy Modalities, Systematic Review, Health Services Accessibility

## Abstract

**Introduction:**

Physiotherapy is a recommended treatment for a wide range of conditions. However, waitlists can delay access to care, leading to poorer patient outcomes and added strain on healthcare systems. Group-based physiotherapy treatments have shown comparable clinical effectiveness to individual care and may help address these challenges. Despite this potential, their implementation in practice remains limited. Therefore, this systematic review aims to: (1) identify the determinants of use, intention to use, satisfaction, attitudes and experience with group-based treatments of patients and physiotherapy professionals, such as physiotherapists or physiotherapy technologists and (2) assess the factors that may influence patients’ and professionals’ preferences for group-based treatments over individual treatments.

**Methods and analysis:**

Systematic searches were conducted on 6 September 2024 in the Medline, Embase, Web of Science, EBSCO CINAHL and Cochrane databases to identify studies published in French or English, in line with the language proficiency of the review team. Eligible studies will use quantitative, qualitative or mixed-method designs and report original data on the use or intention to use, satisfaction with, attitudes toward and experiences with group-based physiotherapy treatments, from either patients or physiotherapy professionals. Studies will be screened by two independent reviewers, with any discrepancy resolved through consensus or by involving a third reviewer. Data will be extracted from the included studies by two independent reviewers using a predefined data extraction form, with any discrepancy similarly resolved. For quantitative findings, the direction of the relationship between the outcomes and their determinants will be reported, along with the magnitude and significance of the coefficients, where available. For qualitative findings, relevant quotes will be provided to illustrate the relationship between the outcomes and their determinants. All included studies will be assessed by two independent reviewers using the Mixed Methods Appraisal Tool, with any discrepancy resolved again through consensus or by involving a third reviewer.

**Ethics and dissemination:**

Ethical approval is not required for this review as it involves the collection of data from existing publications only. The findings will be disseminated through publication in a peer-reviewed scientific journal and presentations at relevant conferences.

**Trial registration number:**

CRD42024574513.

**STRENGTHS AND LIMITATIONS OF THIS STUDY**

- A specialised librarian will validate the search strategies for each database to help ensure a comprehensive and precise literature review.
- The Mixed Methods Appraisal Tool, a research-based instrument adapted to various study designs, will be used to systematically assess the risk of bias in all included studies.
- The review will include both quantitative and qualitative studies and use an integrated approach to synthesise findings across diverse study designs to enrich its findings.
- The inclusion of diverse study designs and data types may introduce complexity in synthesis but allows for a more comprehensive understanding of the topic.
- Heterogeneity across included studies is expected and will preclude the use of meta-analysis.

## Introduction

 Physiotherapy is a recommended treatment for a wide range of conditions, including neurological, musculoskeletal and cardiopulmonary conditions.^1^ However, long waitlists for physiotherapy services limit timely access to care in many countries,^2^,^5^ potentially leading to poorer patient outcomes and prognosis, even after treatment is eventually received.^6^ Moreover, these delays are not evenly distributed and may exacerbate existing socioeconomic inequities.^7^ This issue also places additional pressure on healthcare providers managing waitlists^8^ and further strains already overburdened healthcare systems, which must then address more complex cases and preventable decompensations.^9^

In response to these challenges, several strategies have been proposed, including the use of group-based physiotherapy treatments.^2 10^ Group-based interventions in physiotherapy have shown comparable clinical effectiveness to individual care in achieving various clinical goals across neurological,^11 12^ musculoskeletal^13 14^ and cardiovascular conditions,^15 16^ as well as urinary incontinence.^17^ Additionally, group-based treatments may reduce costs for healthcare systems in publicly funded settings and for patients in private clinics.^13 18^ Beyond alleviating waitlist pressures, group-based treatments could offer additional clinical benefits by fostering peer support and motivation among group members, therefore enhancing treatment adherence,^19^ a key factor in attaining optimal clinical outcomes in physiotherapy.^20 21^

Despite this promising potential, the uptake of group-based treatments in routine physiotherapy practice still remains limited.^2 10^ Although a recent scoping review demonstrated the feasibility and high acceptability of group-based exercise programmes in physiotherapy,^22^ it did not explore the underlying reasons for these outcomes. The barriers to broader implementation appear more complex, suggesting the need for a more indepth and comprehensive investigation into the factors that determine the engagement with such treatments of both patients and physiotherapy professionals, such as physiotherapists or physiotherapy technologists. This systematic review thus aims to address these gaps by: (1) identifying the determinants of patients’ and physiotherapy professionals’ use, intention to use, satisfaction, attitudes and experience with group-based treatments and (2) assessing the factors that may influence their preferences for group-based treatments over individual treatments.

## Methods and analysis

This protocol is reported following the Preferred Reporting Items for Systematic Reviews and Meta-Analyses Protocols^23^ statement and has been officially registered on PROSPERO (CRD42024574513). The systematic review will ultimately inform a discrete choice experiment (DCE) designed to explore key reasons and their relative importance in the use of group-based rehabilitation treatments, from both patients’ and physiotherapy professionals’ perspectives. Its findings will establish the foundation for identifying attributes and levels that will shape the choice sets in upcoming DCE scenarios. Furthermore, this systematic review will also guide the identification of subgroups for further analysis based on patients’ or physiotherapy professionals’ characteristics recognised as determinants or influencing factors.

### Eligibility criteria

#### Types of studies

All studies published in French or English, using quantitative, qualitative or mixed-method designs and reporting original data will be eligible for inclusion in this review, including pilot studies. The language restrictions are based on the language proficiency of the review team, enabling rigorous screening, data extraction and critical appraisal without the need for translation services, thus preserving both methodological rigour and the feasibility of the review process. Editorials, commentaries, blog publications, poster or conference abstracts, study protocols, errata, theses, dissertations, methodology papers and descriptions of interventions without original data will be excluded. While the reference lists of relevant reviews will be screened for additional references, the reviews themselves will be excluded. For any relevant protocols identified, searches will be conducted to find published results. However, the protocols themselves will be excluded.

#### Participants

All studies involving adults and older adults with any health condition, as well as physiotherapy professionals, will be eligible for inclusion, as both perspectives are of interest in this review. Studies focusing on adolescents, children, infants, healthy individuals or other healthcare professionals and non-healthcare professionals will be excluded.

#### Interventions

All studies on group-based treatments provided by one or more physiotherapy professionals as part of a comprehensive rehabilitation programme will be eligible for inclusion, whether the interventions are delivered in an inpatient or ambulatory setting. Interventions must involve direct, synchronous interactions, either in person or via telerehabilitation, and be delivered to groups consisting of two or more individuals receiving care. Studies will be excluded if they involve interventions not delivered by physiotherapy professionals, do not include direct synchronous interactions or focus solely on education, support or lifestyle changes. Additionally, individual-based interventions, supervised open gym spaces without structured treatment (ie, drop-in spaces) and health promotion programmes targeting healthy individuals will be excluded.

#### Outcomes

This review will include studies reporting on the determinants of:

- Use and intention to use: this outcome will capture current and planned future utilisation of group-based physiotherapy treatments, either as a form of care or as a clinical practice. It will provide insights into adoption patterns based on preferences,^24^ reflecting how individuals anticipate engaging with group-based physiotherapy treatments. This outcome is therefore consistent with the DCE’s focus on real-world choices.
- Satisfaction: in health economics, where DCE methodology originates, the concept of ‘utility’ refers to the satisfaction or benefit derived from consuming a service, which guides individual choices and helps establish preferences.^25^ Since a DCE assesses utility by quantifying the expected satisfaction associated with various choices,^24^ measuring satisfaction also appears relevant for this review. In physiotherapy, satisfaction is a multidimensional concept related to the quality of care. From the patient perspective, it extends beyond clinical outcomes to encompass the care environment and interpersonal relationships with healthcare providers.^26^ Satisfaction reflects the evaluation of the care received in relation to expectations.^27^ As higher levels of satisfaction are associated with better treatment adherence, particularly in terms of attendance,^28^ it appears directly related to use and intention to use and will therefore be relevant for this review. From the physiotherapy professionals’ perspective, satisfaction with their role and the provided care can involve factors such as the recognition of skills and experience, autonomy, access to mentoring and peer support and work-life balance, among others.^29^ As job satisfaction can drive the adoption of new clinical practices in physiotherapy,^30^ it also appears related to use and intention to use and will thus be relevant for this review.
- Attitudes: this outcome will encompass affective, behavioural and cognitive responses^31^ to group-based treatments in rehabilitation, either from the patients’ or the physiotherapy professionals’ perspectives. Some authors define attitudes as the tendency to ‘(evaluate) a particular entity with some degree of favour or disfavour’,^25^ which aligns with concepts of choice and preference and thus is consistent with a DCE. Additionally, as a construct, attitudes towards healthcare can sometimes be assessed through more easily measurable variables like satisfaction, preferences or care experience.^32^ Therefore, the attitudes of patients and physiotherapy professionals towards group-based physiotherapy treatments appear closely related to use, intention to use and satisfaction.
- Experience: this outcome will centre on the overall experience of care. Sometimes used synonymously for satisfaction,^24 33^ the experience of care is less about the evaluation of the healthcare encounter and more about what actually happened during that encounter.^34^ From the patients’ perspective, it will mostly describe the interactions with the healthcare system and the physiotherapy professionals throughout group-based treatments.^27^ From the physiotherapy professionals’ perspective, it could describe the provision of group-based treatments.
- Preferences: this outcome reflects potential choice making or willingness to select group-based treatments over other existing care options, highlighting underlying values and desires.^35^ As such, it also appears related to use and intention to use.

### Databases and search strategy

A professional specialised librarian with expertise in search strategies related to physiotherapy and rehabilitation sciences has validated the search strategies, including subject headings and keywords on the concepts of ‘physiotherapy’, ‘group-based treatments’ and ‘determinants of use’ and other relevant variations such as ‘physical therapy’ or ‘rehabilitation’. Searches were conducted on 6 September 2024 in the Medline, Embase, Web of Science, EBSCO CINAHL and Cochrane databases from their inception up until the search date. No specific limits were applied to the search. Reference lists of relevant reviews were screened to identify additional eligible studies. For protocols, the research team verified whether any data from the described studies has been published since their publication. The search strategies for the previously cited databases are available in online supplemental file 1.

### Selection process

Studies will be screened in a two-step process (titles/abstracts and full text) by two independent reviewers. Any discrepancy will be resolved through consensus or by involving a third reviewer as needed. Full texts will be retrieved online using university-affiliated subscriptions or, when not accessible via those resources, by contacting corresponding authors.

### Data collection process

Data will be extracted from the included studies by two independent reviewers using a predefined data extraction form (online supplemental file 2). This form will capture the following information:

- Study characteristics: identification information, study design, objectives and sources of funding.
- Study participants: demographic characteristics, inclusion and exclusion criteria, condition targeted, sample sizes, participant flow and attrition rates, country.
- Intervention or exposure to group-based physiotherapy treatments: duration, description of exercises following relevant items from the Consensus on Exercise Reporting Template (CERT)^36^ and any comparators, if applicable.
- Reported outcomes: whether the perspective reported is from the participants as patients or physiotherapy professionals, use of any theoretical framework, reported relevant outcomes, the measurement tools used and their associated findings for quantitative data or relevant quotes for qualitative data.

Any discrepancy will be resolved through consensus or by involving a third reviewer. The CERT is a 16-item tool, internationally endorsed and specifically designed to guide the reporting of exercise programmes.^36^

### Data synthesis

For quantitative findings, the direction of the relationship between the outcomes and their determinants will be reported, along with the magnitude and significance of the coefficients, where available. For qualitative findings, relevant quotes will be provided to illustrate the relationship between the outcomes and their determinants. Given the expected heterogeneity across studies in terms of design, populations and outcomes, a narrative synthesis will be conducted. Heterogeneity will be explored descriptively by examining variations in study characteristics, study participants, interventions and reported outcomes. When appropriate and if sufficient data are available, subgroup analyses will be conducted to explore patterns among studies that share similar characteristics.

### Risk of bias assessment

All included studies will be assessed by two independent reviewers using the latest version of the Mixed Methods Appraisal Tool (MMAT).^37^ Again, any discrepancy will be resolved through consensus or by involving a third reviewer as needed. The MMAT is a tool frequently used in systematic reviews, grounded in the literature, that offers criteria specific to various study designs, including quantitative, qualitative and mixed designs. The MMAT includes five methodological quality criteria tailored to five categories of study designs, allowing a structured assessment of potential biases relevant to each type.^37^ If major biases are identified through this process, the research team will conduct sensitivity analyses excluding the affected studies to explore their impact on the overall findings.

### Patient and public involvement

Patients were not involved in the design or conception of this systematic review protocol.

## Ethics and dissemination

As systematic reviews are based on existing data from published articles, formal ethical approval is not required for this study.

The findings from this systematic review will be submitted for publication in a peer-reviewed scientific journal and presented at relevant conferences. They will also inform a DCE aimed at both patients and physiotherapy professionals by guiding the development of choice sets through the identification of key attributes and levels. This future DCE will additionally incorporate insights from an upcoming qualitative study, with the findings from both this systematic review and the qualitative study integrated to finalise the scenarios.

## Discussion

This systematic review will provide relevant insights into the field of physiotherapy by shedding light on the factors influencing the engagement of both patients and physiotherapy professionals with group-based physiotherapy treatments, which have been proposed as a promising solution to reduce long waiting times.^2 10^ Indeed, despite their demonstrated clinical effectiveness^11^,^17^ and potential to alleviate healthcare system burdens,^13 18^ group-based interventions have remained underutilised. This review will therefore provide key insights that could inform strategies to enhance their uptake and potentially improve timely access to care.

This review may, however, be limited by the heterogeneity of the included studies, which precludes the conduct of a meta-analysis. Nonetheless, the inclusion of both quantitative and qualitative data is expected to enhance the comprehensiveness and depth of the synthesis. Despite the use of a rigorous and comprehensive search strategy across multiple databases, it remains possible that relevant studies published in sources not indexed in these databases or within the grey literature may have been missed.

The findings from this review could also provide considerations for practice and policy. Understanding the determinants of use, intention to use, satisfaction, attitudes and experience of group-based treatments could guide future implementation efforts. Moreover, by informing the development of a DCE, this review will support a deeper investigation into preferences for group-based physiotherapy treatments. Together, this process could contribute to reducing wait times and associated consequences, in order to promote more accessible physiotherapy services.

## Supplementary material

10.1136/bmjopen-2024-095201 online supplemental file 1
