# Supplementary material for: Patients and physiotherapy professionals’ perspectives on group-based treatments: a systematic review protocol
Source: BMJ Open. 2025 Jun 3;15(6):e095201. doi: 10.1136/bmjopen-2024-095201 (PMC12142124; doi:10.1136/bmjopen-2024-095201)
Supplement: online supplemental file 1 [file bmjopen-15-6-s001.docx]

**Online supplemental files**

***Online supplemental file 1. Search strategies for the different selected databases***

| **Databases** | **Search lines** | **Search terms** |
| --- | --- | --- |
| Medline | 1 | (physiotherap* or "physical therap*" or "exercise therap*" or "therapeutic exercis*" or rehabilitation or telerehabilitation).ti,ab,kf. |
|  | 2 | Physical Therapy Modalities/ or Exercise Therapy/ or Rehabilitation/ or Cardiac Rehabilitation/ or Neurological Rehabilitation/ or Telerehabilitation/ or exp Physical Therapy Specialty/ or exp Physical therapists/ |
|  | 3 | 1 or 2 |
|  | 4 | (group-based or "group-based" or "group exercis*" or group-exercis* or "group treatment*" or "group structure*" or "group setting" or "peer group*" or "group dynamic*").ti,ab,kf. |
|  | 5 | exp Group Structure/ or exp Peer Group/ or Group Dynamics/ |
|  | 6 | 4 or 5 |
|  | 7 | (preference* or "determinant* of use" or "social determinant*" or associat* or relationship* or factor* or predictor* or "treatment adherence" or "treatment compliance" or accept* or satisf* or usage or "use" or utilisation or utilization or perception* or perceive* or adopt* or experience* or behavio* or attitud*).ti,ab,kf. |
|  | 8 | exp Social Determinants of Health/ or Health Behavior/ or "treatment adherence and compliance"/ or exp Patient Preferences/ or exp Patient Acceptance of Health Care/ or exp Socioeconomic Factors/ or exp Health Knowledge, Attitudes, Practice/ |
|  | 9 | 7 or 8 |
|  | 10 | 3 and 6 and 9 |
| Embase | 1 | (physiotherap* or "physical therap*" or "exercise therap*" or "therapeutic exercis*" or rehabilitation or telerehabilitation).ti,ab,kf. |
|  | 2 | exp physiotherapy/ or physiotherapy practice/ or rehabilitation/ or athletic rehabilitation/ or cancer rehabilitation/ or geriatric rehabilitation/ or heart rehabilitation/ or neurorehabilitation/ or pulmonary rehabilitation/ or rehabilitation care/ or telerehabilitation/ |
|  | 3 | 1 or 2 |
|  | 4 | (group-based or "group-based" or "group exercis*" or group-exercis* or "group treatment*" or "group structure*" or "group setting" or "peer group*" or "group dynamic*").ti,ab,kf. |
|  | 5 | exp Group Structure/ or exp Peer Group/ or Group Dynamics/ |
|  | 6 | 4 or 5 |
|  | 7 | (preference* or "determinant* of use" or "social determinant*" or associat* or relationship* or factor* or predictor* or "treatment adherence" or "patient compliance" or accept* or satisf* or usage or "use" or utilisation or utilization or perception* or perceive* or adopt* or experience* or behavio* or attitud*).ti,ab,kf. |
|  | 8 | Social Determinants of Health/ or Health Behavior/ or Patient compliance/ or exp Patient Preference/ or exp Patient Attitude/ or Health Personnel Attitude/ or Physiotherapist Attitude/ |
|  | 9 | 7 or 8 |
|  | 10 | 3 and 6 and 9 |
| Web of Science | 1 | ALL=(physiotherap* OR "physical therap*" OR "exercise therap*" OR "therapeutic exercis*" OR rehabilitation OR telerehabilitation) |
|  | 2 | ALL=(group-based OR "group-based" OR "group exercis*" OR group-exercis* OR "group treatment*" OR "group structure*" OR "group dynamic*" OR "group setting" OR "peer group*") |
|  | 3 | ALL=(preference* OR "determinant* of use" OR "social determinant*" OR associat* OR relationship* OR factor* OR predictor* OR "treatment adherence" OR "treatment compliance" OR accept* OR satisf* OR usage OR "use" OR utilisation OR utilization OR perception* OR perceive* OR adopt* OR experience* OR behavio* OR attitud*) |
|  | 4 | #3 AND #2 AND #1 |
| CINAHL | S1 | TI (physiotherap* OR "physical therap*" OR "exercise therap*" OR "therapeutic exercis*" OR rehabilitation OR telerehabilitation) OR AB (physiotherap* OR "physical therap*" OR "exercise therap*" OR "therapeutic exercis*" OR rehabilitation OR telerehabilitation) |
|  | S2 | (MH "Rehabilitation") OR (MH "Therapeutic Exercise") OR (MH "Rehabilitation, Cancer") OR (MH "Rehabilitation, Pulmonary") OR (MH "Rehabilitation Patients") OR (MH "Rehabilitation, Geriatric") OR (MH "Rehabilitation, Cardiac") OR (MH "Physical Therapy") OR (MH "Physical Therapists") OR (MH "Physical Therapist Attitudes") OR (MH "Physical Therapist Assistants") OR (MH "Physical Therapy Service") |
|  | S3 | S1 OR S2 |
|  | S4 | TI ("group-based" OR "group exercis*" OR "group-exercis*" OR "group dynamics" OR "peer group*" OR "group setting" OR "group treatment*") OR AB ("group-based" OR "group exercise*" OR "group-exercis*" OR "group dynamic*" OR "peer group*" OR "group setting" OR "group treatment*") |
|  | S5 | (MH "Group Exercise") OR (MH "Group Dynamics") OR (MH "Peer Group") |
|  | S6 | S4 OR S5 |
|  | S7 | TI ("preference*" OR "determinant* of use" OR "social determinant*" OR "associat*" OR "relationship*" OR "factor*" OR "predictor*" OR "accept*" OR "satisf*" OR "usage" OR "use" OR "utilisation" OR "utilization" OR "perception*" OR "perceive*" OR "adopt*" OR "experience*" OR "behavio*" OR "attitud*") OR AB ("preference*" OR "determinant* of use" OR "social determinant*" OR "associat*" OR "relationship*" OR "factor*" OR "predictor*" OR "accept*" OR "satisf*" OR "usage" OR "use" OR "utilisation" OR "utilization" OR "perception*" OR "perceive*" OR "adopt*" OR "experience*" OR "behavio*" OR "attitud*") |
|  | S8 | (MH "Health Resource Utilization") OR (MH "Socioeconomic Factors") OR (MH "Sociodemographic Factors") OR (MH "Perception") OR (MH "Social Determinants of Health") OR (MH "Patient Preference") OR (MH "Physical Therapist Attitudes") OR (MH "Patient Attitudes") |
|  | S9 | S7 OR S8 |
|  | S10 | S3 AND S6 AND S9 |
| Cochrane | 1 | physiotherap* OR "physical therap*" OR "exercise therap*" OR "therapeutic exercis*" OR rehabilitation OR telerehabilitation |
|  | 2 | group-based OR "group-based" OR "group exercis*" OR group-exercis* OR "group treatment*" OR "group structure*" OR "group dynamic*" OR "group setting" OR "peer group*" |
|  | 3 | preference* OR "determinant* of use" OR "social determinant*" OR associat* OR relationship* OR factor* OR predictor* OR "treatment adherence" OR "treatment compliance" OR accept* OR satisf* OR usage OR "use" OR utilisation OR utilization OR perception* OR perceive* OR adopt* OR experience* OR behavio* OR attitud* |
|  | 4 | #1 AND #2 AND #3 |

***Online supplemental file 2. Data extraction form***

| **Study characteristics** | Study ID |  |
| --- | --- | --- |
|  | First author |  |
|  | Publication year |  |
|  | Title |  |
|  | Journal |  |
|  | Companion paper or related publications (if any) |  |
|  | Study design |  |
|  | Study objectives/aims |  |
|  | Funding sources |  |
| **Sample and setting** | Mean age/Age criteria |  |
|  | Sex distribution |  |
|  | Inclusion/Exclusion criteria |  |
|  | Condition targeted |  |
|  | Sample size |  |
|  | Withdrawal/Dropout rate |  |
|  | Reported reasons for withdrawal/dropout |  |
|  | Country |  |
| **Rehabilitation program description** | Rehabilitation program duration |  |
|  | Physiotherapy professional delivering the rehabilitation program |  |
|  | Recruitment process for the rehabilitation program (ex. doctor’s referral) |  |
|  | Exercise equipment (CERT item #1) |  |
|  | Description of expertise or training of the physiotherapy professional delivering the rehabilitation program (CERT item #2) |  |
|  | Group size within the rehabilitation program |  |
|  | Description of delivery or level of supervision (CERT item #4) |  |
|  | Description of reported motivation strategies (CERT item #6) |  |
|  | Description of how the exercise progression was determined (CERT item #7) |  |
|  | Description of the exercises within the rehabilitation program (CERT item #8) |  |
|  | Description of any home-based individual component in addition to the group-based component (CERT item #9) |  |
|  | Description of any non-exercise components (CERT item #10) |  |
|  | Description of the setting of the rehabilitation program (CERT item #12) |  |
|  | Generic or tailored (CERT item #14) |  |
|  | Description of any levels within the rehabilitation program (CERT item #15) |  |
|  | Description of any remote component of the rehabilitation program |  |
| **Reported outcomes** | Perspectives reported |  |
|  | Description of any theoretical framework or model used |  |
|  | Reported outcomes |  |
|  | Measurement used to report each outcomes |  |
|  | Analyses used |  |
|  | Statistical tools used for the analyses |  |
|  | Main reported findings for each outcomes |  |
