# Supplementary material for: Patients and physiotherapy professionals’ perspectives on group-based treatments: a systematic review protocol
Source: BMJ Open. 2025 Jun 3;15(6):e095201. doi: 10.1136/bmjopen-2024-095201 (PMC12142124; doi:10.1136/bmjopen-2024-095201)
Supplement: Supplementary file 2 [file bmjopen-2024-095201.reviewer_comments.pdf]

PEER REVIEW HISTORY

BMJ Open publishes all reviews undertaken for accepted manuscripts. Reviewers are asked to complete a checklist review form and are provided with free text boxes to elaborate on their assessment. These free text comments are reproduced below.

ARTICLE DETAILS

Title (Provisional)

Patients and physiotherapy professionals' perspectives on group-based treatments: a systematic review protocol

Authors

Le Berre, Mélanie; Laberge, Maude; Yousefi, Farzaneh

VERSION 1 - REVIEW

**Reviewer**	**1**
**Name**	**Otón, Teresa**
**Affiliation**	**Instituto de Salud Musculoesquelética SL**
**Date**	**11-Dec-2024**
**COI**	**None**

Title and Abstract

The title appropriately identifies the report as a systematic review protocol. The abstract provides a structured summary of the key elements, including background, objectives, methods, and planned dissemination. To fully comply with PRISMA, consider adding:

Eligibility criteria for participants and interventions

Information sources (e.g., databases to be searched)

Risk of bias assessment method

Introduction

The introduction provides a clear rationale for the review and explicit objectives. It aligns well with PRISMA guidelines.

Methods

Eligibility Criteria

The eligibility criteria are well-defined for study designs, participants, interventions, and outcomes. To enhance compliance with PRISMA:

Clarify the rationale for these language restrictions

Information Sources

The protocol lists the databases to be searched but could be improved by:

Specifying the dates of coverage for each database

Mentioning any planned hand-searching of reference lists or contacting experts

Search Strategy

The full search strategy is provided in the supplementary material, which is appropriate. Consider:

Briefly describing the key search terms in the main text

Mentioning any limits applied to the search (e.g., date restrictions)

Study Selection and Data Extraction

The process for study selection and data extraction is well-described. To fully align with PRISMA:

Specify how disagreements between reviewers will be resolved (e.g., discussion, third reviewer)

Describe the process for obtaining full-text articles

Data Items

The data items to be extracted are comprehensively listed in the supplementary material. In the main text:

Provide a summary of the key data items to be collected

Risk of Bias Assessment

The use of the Mixed Methods Appraisal Tool (MMAT) is mentioned. To improve:

Briefly describe the key domains of the MMAT

Explain how the results of the risk of bias assessment will be used in the synthesis

Data Synthesis

The planned approach to data synthesis is described. To enhance:

Clarify how heterogeneity between studies will be assessed and addressed

Specify any planned subgroup or sensitivity analyses

Discussion

The discussion section outlines the potential implications of the review. To fully align with PRISMA:

Discuss potential limitations of the review methodology

Mention plans for updating the review, if any

Other Information

The protocol registration is appropriately mentioned.

**Reviewer**	**2**
**Name**	**Treacy, Daniel**
**Affiliation**	**South Eastern Sydney Local Health District, Physiotherapy**
**Date**	**09-Apr-2025**
**COI**	**None**

Title - Needs to include in title that's a protocol for a systematic review - otherwise readers will assume its the actual SR

Line 32, page 2 - suggest change 'their' to 'physiotherapy professionals' however abstract only mentions Physiotherapy professionals - please update

Line 58, page 3 - I wouldn't say this was a strength of the study

Line 9, page 4 - go into more detail how this will be different to this scoping review (22)

Line 128, page 7 - Will this include interventions delivered both in the hospital setting as well as in the community. Please include in the manuscript

Line 182 pg 11 - Rehabilitation means different things to many people. For some its a specialised type of physiotherapy, others its a specialised type of services delivered under a medical rehabilitation consultant and for others - its providing exercise to recover. I think its a bit confusing to use in regards to a librarian

Line 243, page 11- you mention that will shed light influencing 'patients'

VERSION 1 - AUTHOR RESPONSE

Reviewer 1's comments: Dr. Teresa Otón, Instituto de Salud Musculoesquelética SL

__**Comment #10:**__
Title and Abstract
The title appropriately identifies the report as a systematic review protocol. The abstract provides a structured summary of the key elements, including background, objectives, methods, and planned dissemination. To fully comply with PRISMA, consider adding:
Eligibility criteria for participants and interventions
Information sources (e.g., databases to be searched)
Risk of bias assessment method

__Response to Comment #10:__
Thank you for your suggestion, and we appreciate your input. The eligibility criteria, information sources, and risk of bias assessment methods are included in the abstract, although they are not under

a specific subtitle. While we believe this format is sufficient to provide the necessary details, we have revised the wording for clarity.
The eligibility criteria for participants are "patients or physiotherapy professionals" (page 2, line 39).
We have specified the eligibility criteria for interventions:

- Page 2, lines 38-39 (added text in bold): “group-based **physiotherapy** treatments”

The information sources include "Medline, Embase, Web of Science, EBSCO CINAHL, and Cochrane databases" (page 2, line 35).
The risk of bias assessment method is described as: "All included studies will be assessed by two independent reviewers using the Mixed Methods Appraisal Tool, with any discrepancy resolved through consensus or by involving a third reviewer" (pages 2-3, lines 46-48).

Comment #11:
Introduction
The introduction provides a clear rationale for the review and explicit objectives. It aligns well with PRISMA guidelines.

Response to Comment #11:
Thank you for this positive feedback. We appreciate your careful review of the manuscript.

Comment #12:
Methods
Eligibility Criteria
The eligibility criteria are well-defined for study designs, participants, interventions, and outcomes.
To enhance compliance with PRISMA:
Clarify the rationale for these language restrictions

Response to Comment #12:
Thank you for this comment. We have clarified the rationale for including only studies published in French or English. These language restrictions reflect the language competencies of the review team, which allow for rigorous screening, extraction, and critical appraisal without the need for translation services. This approach ensures the accuracy and reliability of the data analysis while maintaining feasibility for the review process. We have updated the manuscript accordingly

- Page 2, lines 34-36 (added text in bold): Systematic searches will be conducted in the Medline, Embase, Web of Science, EBSCO CINAHL, and Cochrane databases to identify studies published in French or English, **in line with the language proficiency of the review team**.
- Page 7, lines 120-124 (added text in bold): All studies published in French or English, using quantitative, qualitative, or mixed-method designs and reporting original data will be eligible for inclusion in this review, including pilot studies. **The language restrictions are based on the language proficiency of the review team, enabling rigorous screening, data extraction, and critical appraisal without the need for translation services, thus preserving both methodological rigour and the feasibility of the review process.**

Comment #13:
Information Sources
The protocol lists the databases to be searched but could be improved by:
Specifying the dates of coverage for each database
Mentioning any planned hand-searching of reference lists or contacting experts

Response to Comment #13:

Thank you for your feedback. As suggested, we have now included the specific search date in both the abstract and the main text to clarify the upper limit of the coverage period. The start date was already indicated as the inception date of each database:

- Page 2, lines 34-36 (added text in bold): Methods and analysis: Systematic searches **were conducted on September 6th, 2024** in the Medline, Embase, Web of Science, EBSCO CINAHL, and Cochrane databases to identify studies published in French or English**, in line with the language proficiency of the review team**.
- Page 10, lines 196-197 (added text in bold): Searches **were conducted on September 6th, 2024** in the Medline, Embase, Web of Science, EBSCO CINAHL, and Cochrane databases from their inception up until the search date.

Also, although reviews and protocols were excluded from the analysis, they were used to support additional handsearches when relevant. Specifically, the reference lists of included reviews were screened to identify eligible primary studies, and for protocols, we verified whether any data had been published since their initial registration. We have clarified this process in the main text:

- Page 10, lines 198- 201 (added text in bold): **Reference lists of relevant reviews were screened to identify additional eligible studies. For protocols, the research team verified whether any data from the described studies has been published since their publication**. The search strategies for the previously cited databases are available in online supplemental file 1.

Comment #14:

Search Strategy

The full search strategy is provided in the supplementary material, which is appropriate. Consider:

Briefly describing the key search terms in the main text

Mentioning any limits applied to the search (e.g., date restrictions)

Response to Comment #14:

Thank you for your suggestion regarding the clarity of the search strategy. We now provide a brief example of variants of the key search terms to enhance transparency and offer a more detailed description of the search strategy in the main text. We also clarified that no additional limits were applied beyond the predefined eligibility criteria, which are already described in the manuscript:

- Page 10, lines 192- 198 (added text in bold): A professional **specialized** librarian **with expertise in search strategies related to physiotherapy and rehabilitation sciences** has validated the search strategies, including subject headings and keywords on the concepts of 'physiotherapy', 'group-based treatments' and 'determinants of use' and other relevant variations **such as 'physical therapy' or 'rehabilitation'**. Searches **were** conducted **on September 6th, 2024** in the Medline, Embase, Web of Science, EBSCO CINAHL, and Cochrane databases from their inception up until the search date. **No specific limits were applied to the search**.

Comment #15:

Study Selection and Data Extraction

The process for study selection and data extraction is well-described. To fully align with PRISMA:

Specify how disagreements between reviewers will be resolved (e.g., discussion, third reviewer)

Describe the process for obtaining full-text articles

Response to Comment #15:

Thank you for your comment. As noted, the manuscript already specifies how disagreements between reviewers will be resolved, which aligns with PRISMA guidelines. We revised it to enhance clarity. Regarding the process for obtaining full-text articles, while this level of detail is not commonly reported in systematic review protocols, , we agree that this information can enhance transparency. We have therefore added a brief explanation:

- Page 10, lines 205-207 (added text in bold): Any discrepancy will be resolved through consensus or by involving a third reviewer **as needed**. **Full texts will be retrieved online using university-affiliated subscriptions or, when not accessible via those resources, by contacting corresponding authors.**

Comment #16:
Data Items
The data items to be extracted are comprehensively listed in the supplementary material. In the main text:
Provide a summary of the key data items to be collected

Response to Comment #16:
Thank you for your comment. To maintain clarity and avoid redundancy, we have opted to present a concise overview of the main categories of data to be extracted directly in the manuscript. These categories are described in the Data Collection Process section, which we believe offers a sufficient level of detail for readers:

- Page 11, lines 210-222: Data will be extracted from the included studies by two independent reviewers using a predefined data extraction form (online supplemental file 2). This form will capture the following information:
    - Study characteristics: identification information, study design, objectives, and sources of funding;
    - Study participants: demographic characteristics, inclusion and exclusion criteria, condition targeted, sample sizes, participant flow, and attrition rates, country;
    - Intervention or exposure to group-based physiotherapy treatments: duration, description of exercises following relevant items from the Consensus on Exercise Reporting Template (CERT)36, and any comparators, if applicable;
    - Reported outcomes: whether the perspective reported is from the participants are patients or physiotherapy professionals, use of any theoretical framework, reported relevant outcomes, the measurement tools used and their associated findings for quantitative data, or relevant quotes for qualitative data.

For full transparency, the complete list of specific items to be extracted within these categories is available in the data extraction form included as online supplemental file 2. We believe this approach aligns with reporting standards and ensures accessibility of all relevant information without overloading the main text.

Comment #17:
Risk of Bias Assessment
The use of the Mixed Methods Appraisal Tool (MMAT) is mentioned. To improve:
Briefly describe the key domains of the MMAT
Explain how the results of the risk of bias assessment will be used in the synthesis

Response to Comment #17:
Thank you for your comment. While the MMAT is a widely used and well-established tool, we have further expanded our description in the manuscript to include its key features for clarity and completeness. We also clarified how the results of the risk of bias assessment will inform the synthesis:

- Page 12, lines 240-247 (added text in bold): Again, any discrepancy will be resolved through consensus or by involving a third reviewer **as needed**. The MMAT is a tool frequently used in systematic reviews, grounded in the literature, that offers criteria specific to various study

designs, including quantitative, qualitative, and mixed designs. **The MMAT includes five methodological quality criteria tailored to five categories of study designs, allowing a structured assessment of potential biases relevant to each type.**[37] **If major biases are identified through this process, the research team will conduct sensitivity analyses excluding the affected studies to explore their impact on the overall findings.**

Comment #18:

Data Synthesis

The planned approach to data synthesis is described. To enhance:

Clarify how heterogeneity between studies will be assessed and addressed

Specify any planned subgroup or sensitivity analyses

Response to Comment #18:

Thank you for this helpful suggestion. We have expanded the description of our planned data synthesis approach to more explicitly address heterogeneity and potential subgroup analyses:

- Pages 11-12, lines 231-236 (added text in bold): **Given the expected heterogeneity across studies in terms of design, populations, and outcomes, a narrative synthesis will be conducted. Heterogeneity will be explored descriptively by examining variations in study characteristics, study participants, interventions and reported outcomes. When appropriate and if sufficient data are available, subgroup analyses will be conducted to explore patterns among studies that share similar characteristics.**

Comment #19:

Discussion

The discussion section outlines the potential implications of the review. To fully align with PRISMA:

Discuss potential limitations of the review methodology

Mention plans for updating the review, if any

Response to Comment #19:

Thank you for providing us with this feedback. We have expanded the discussion section to acknowledge potential limitations of the review methodology:

- Pages 13-14, lines 275-280 (added text in bold): **This review may however be limited by the heterogeneity of the included studies, which precludes the conduct of a meta-analysis. Nonetheless, the inclusion of both quantitative and qualitative data is expected to enhance the comprehensiveness and depth of the synthesis. Despite the use of a rigorous and comprehensive search strategy across multiple databases, it remains possible that relevant studies published in sources not indexed in these databases or within the grey literature may have been missed.**

Regarding updates of the review, the PRISMA-P mentions "If the protocol is for an update of a previous systematic review, identify as such" but does not require forecasting future updates. As such, no plan for future updates has been included at this stage.

Comment #20:

Other Information

The protocol registration is appropriately mentioned.

Response to Comment #20:

Thank you for highlighting this.

Reviewer 2's comments: Mr. Daniel Treacy, South Eastern Sydney Local Health District

Comment #21:
Title - Needs to include in title that's a protocol for a systematic review - otherwise readers will assume its the actual SR

Response to Comment #21:
Thank you for your comment. We have added this precision in the title:
- Page 1, lines 1-2 (added text in bold): Patients and physiotherapy professionals' perspectives on group-based treatments: a systematic review **protocol**

Comment #22:
Line 32, page 2 - suggest change 'their' to 'physiotherapy professionals' however abstract only mentions Physiotherapy professionals - please update

Response to Comment #22:
Thank you for this helpful suggestion. We have revised the text to clarify that the review focuses specifically on both patients and physiotherapy professionals, and we have removed references to assistants from the target population accordingly. We also reworded the sentence to make it explicit that the review includes both patients and physiotherapy professionals. The abstract now consistently refers to both populations, enhancing clarity for BMJ Open readers:
- Page 2, lines 29-33 (added text in bold): Therefore, this systematic review aims to 1) identify the determinants of use, intention to use, satisfaction, attitudes, and experience with group-based treatments of patients and physiotherapy professionals such as physiotherapists or physiotherapy technologists, and 2) assess the factors that may influence **patients' and professionals'**preferences for group-based treatments over individual treatments.

Comment #23:
Line 58, page 3 - I wouldn't say this was a strength of the study

Response to Comment #23:
Thank you for your comment. We acknowledge that the involvement of a specialized librarian in validating the search strategies may not constitute a major methodological strength on its own. However, we consider it an important aspect of ensuring a rigorous and transparent systematic review process, particularly in minimizing the risk of missing relevant studies. That said, we have revised the wording to better reflect its supporting role rather than presenting it as a key strength:
- Page 3, lines 59-60 (added tex in bold): A specialized librarian will validate the search strategies for each database to **help** ensure a comprehensive and precise literature review.

Comment #24:
Line 9, page 4 - go into more detail how this will be different to this scoping review (22)

Response to Comment #24:
Thank you for this comment. We have clarified the distinction between the existing scoping review and the objectives of the present systematic review. While the scoping review (reference 22) examined the feasibility and acceptability of group-based exercise programs in physiotherapy, it did not seek to explain *why* these interventions are or are not adopted. Our review then goes further by aiming to identify the determinants – that is, the underlying factors influencing use, intention to use,

satisfaction, attitudes, experiences, and preferences – among both patients and physiotherapy professionals.

This more in-depth approach will help to identify the conditions under which group-based treatments are more likely to be adopted, accepted, or preferred. It also provides insight into the mechanisms and contextual elements that may facilitate or hinder their implementation, thereby informing more targeted and effective implementation strategies for group-based treatments in physiotherapy.

We have revised the manuscript accordingly to better reflect this distinction:

- Pages 5-6, lines 96-105 (added text in bold): Although a recent **scoping** review has demonstrated the feasibility and high acceptability of group-based exercise programs in physiotherapy,[22] **it did not explore the underlying reasons for these outcomes. The** barriers to broader implementation appear more complex**, suggesting the** need for a more **in-depth and** comprehensive investigation into the factors **that determine** the engagement with such treatments of both patients and physiotherapy professionals, such as physiotherapists or, physiotherapy technologists or physiotherapy assistants. This systematic review thus aims to address these gaps by 1) identifying the determinants of patients' and physiotherapy professionals' use, intention to use, satisfaction, attitudes, and experience with group-based treatments and 2) assessing the factors that may influence their preferences for group-based treatments over individual treatments.

<u>**Comment #25:**</u>
Line 128, page 7 - Will this include interventions delivered both in the hospital setting as well as in the community. Please include in the manuscript

<u>Response to Comment #25:</u>
Thank you for your question. Yes, the review will include interventions delivered in both hospital and community settings, as there are no exclusions based on the setting. We have updated the manuscript to reflect this clarification:

- Page 7, lines 138-140 (added text in bold): All studies on group-based treatments provided by one or more physiotherapy professionals as part of a comprehensive rehabilitation program will be eligible for inclusion**, whether the interventions are delivered in an inpatient or ambulatory setting**.

<u>**Comment #26:**</u>
Line 182 pg 11 - Rehabilitation means different things to many people. For some its a specialised type of physiotherapy, others its a specialised type of services delivered under a medical rehabilitation consultant and for others - its providing exercise to recover. I think its a bit confusing to use in regards to a librarian

<u>Response to Comment #26:</u>
Thank you for this valuable comment. We appreciate your point about the possible interpretations of the term "specialized in rehabilitation". The librarian involved in this project is not a clinician but is affiliated with the university's programs in rehabilitation and health sciences, which include physiotherapy, occupational therapy, kinesiology, speech-language pathology, audiology, and biomedical sciences. To avoid confusion, we have revised the manuscript to more accurately describe their expertise and role:

- Page 10, line 192-195 (added text in bold): A professional **specialized** librarian **with expertise in search strategies related to physiotherapy and rehabilitation sciences** has validated the

search strategies, including subject headings and keywords on the concepts of 'physiotherapy', 'group-based treatments' and 'determinants of use' and other relevant variations **such as 'physical therapy' or 'rehabilitation'**.

Comment #27:

Line 243, page 11- you mention that will shed light influencing 'patients'

Response to Comment #27:

Yes, this review aims to shed light on the determinants of use, intention to use, satisfaction, attitudes, and experience, as well as preferences among both patients and physiotherapy professionals. This focus was clarified in response to Comment #22. We have also reworded the sentence in the Discussion to enhance clarity and improve understanding:

- Page 13, lines 267-270 (added text in bold): This systematic review will provide relevant insights into the field of physiotherapy by shedding light on the factors influencing **the engagement of** both patients and physiotherapy professionals with group-based physiotherapy treatments, which have been proposed as a promising solution to reduce long waiting times.[2,10]
